# Social Vulnerability Index and Cognitive Decline in a Population‐Based Cohort Study

**DOI:** 10.1002/alz.089070

**Published:** 2025-01-09

**Authors:** Pankaja Desai, Ted K.S. Ng, Kristin R Krueger, Denis A Evans, Kumar B Rajan

**Affiliations:** ^1^ Rush Institute for Healthy Aging, Chicago, IL USA; ^2^ University of California Davis, Davis, CA USA

## Abstract

**Background:**

Social determinants of health (SDOH) are critically important to reduce disparities in Alzheimer’s disease (AD). Yet, we often focus on individual‐level characteristics, excluding socio‐environmental factors, when evaluating predictors of AD or developing interventions that target AD prevention. To address these gaps, we examine the association between the social vulnerability index (SVI) and cognitive decline in a bi‐racial cohort of older adults.

**Method:**

In this investigation, we use data from participants (N = 6,763) in the Chicago Health and Aging Project (CHAP), a longitudinal, population‐based cohort study in four south‐side Chicago communities. The study sample consists of participants who completed at least two neuropsychological assessments approximately three years apart. SVI is a score which utilizes US Census data to measure certain SDOH and calculates percentile rankings by census tract. Linear mixed effects regression models examined the association between SVI and cognitive decline, adjusting for age, sex, race, and education.

**Result:**

The study sample comprised of more AA (63%) and female (61%) participants. The greatest percentage of AA participants were represented in quartile 3, and the greatest percentage of White participants were represented in quartile 1. We found that participants living in communities with greater SVI had more rapid global cognitive decline (quartile 2 (β = ‐0.010, SE = 0.005, p = 0.05), quartile 3 (β = ‐0.010, SE = 0.004 p = 0.024), and quartile 4 (β = ‐0.014, SE = 0.005, p = 0.005) compared to participants living in the lowest SVI communities.

**Conclusion:**

Social vulnerability plays a crucial role in faster cognitive decline, thereby increasing AD risk in more vulnerable areas. Reducing social vulnerability in communities might help reduce the future risk of AD.

